# Impact of transpulmonary thermodilution-based cardiac contractility and extravascular lung water measurements on clinical outcome of patients with Takotsubo cardiomyopathy after subarachnoid hemorrhage: a retrospective observational study

**DOI:** 10.1186/s13054-014-0482-4

**Published:** 2014-08-12

**Authors:** Tatsushi Mutoh, Ken Kazumata, Shunsuke Terasaka, Yasuyuki Taki, Akifumi Suzuki, Tatsuya Ishikawa

**Affiliations:** Department of Surgical Neurology, Research Institute for Brain and Blood Vessels-AKITA, 6-10 Senshu-Kubota-machi, Akita, 010-0874 Japan; Department of Nuclear Medicine and Radiology, Institute of Development, Aging and Cancer, Tohoku University, 4-1 Seiryo-machi, Aoba-ku, Sendai, 980-8575 Japan; Department of Neurosurgery, Hokkaido University Graduate School of Medicine, North 15, West 7, Kita-ku, Sapporo, 060-8638 Japan; Department of Neurosurgery, Teine Keijinkai Hospital, 1-40 Maeda 1-jo 12-chome, Teine-ku, Sapporo, 006-8555 Japan

## Abstract

**Introduction:**

Takotsubo cardiomyopathy (TCM) is a life-threatening systemic consequence early after subarachnoid hemorrhage (SAH), but precise hemodynamics and related outcomes have not been studied. The purpose of this study was to investigate TCM-induced cardiac function by transpulmonary thermodilution and its impact on clinical outcome of SAH.

**Methods:**

We retrospectively analyzed 46 consecutive postoperative SAH patients who developed TCM. Patients were divided into two groups of echocardiographic left ventricular ejection fraction (LVEF) <40% (TCM with left ventricular (LV) dysfunction) and LVEF ≥40% (TCM without LV dysfunction). Cardiac function index (CFI) and extravascular lung water index (ELWI) were monitored by transpulmonary thermodilution in parallel with serial measurements of echocardiographic parameters and blood biochemical markers.

**Results:**

Transpulmonary thermodilution-derived CFI was significantly correlated with LVEF (*r* = 0.82, *P* < 0.0001). The CFI between days 0 and 7 was significantly lower in patients with LV dysfunction (LVEF <40%) than in patients with LVEF ≥40% (*P* < 0.05). CFI had a better ability than cardiac output to detect cardiac dysfunction (LVEF <40%) (area under the curve = 0.85 ± 0.02; *P* < 0.001). A CFI value <4.2 min^−1^ had a sensitivity of 82% and specificity of 84% for detecting LVEF <40%. CFI <4.2 min^−1^ was associated with delayed cerebral ischemia (DCI) (odds ratio (OR) = 2.14, 95% confidence interval (CI) = 1.33 to 2.86; *P* = 0.004) and poor 3-month functional outcome on a modified Rankin Scale of 4 to 6 (OR = 1.87, 95% CI = 1.06 to 3.29; *P* = 0.02). An extravascular lung water index (ELWI) >14 ml/kg after day 4 increased the risk of poor functional outcome at 3-month follow-up (OR = 2.10, 95% CI = 1.11 to 3.97; *P* = 0.04).

**Conclusions:**

Prolonged cardiac dysfunction and pulmonary edema increased the risk of DCI and poor 3-month functional outcome in postoperative SAH patients with TCM. Serial measurements of CFI and ELWI by transpulmonary thermodilution may provide an easy bedside method of detecting early changes in cardiopulmonary function to direct proper post-SAH treatment.

**Electronic supplementary material:**

The online version of this article (doi:10.1186/s13054-014-0482-4) contains supplementary material, which is available to authorized users.

## Introduction

Autonomic storm after aneurysmal subarachnoid hemorrhage (SAH) can adversely affect cardiovascular parameters and may result in stress-related cardiomyopathy [1]. One distinct morphological variant of this phenomenon is apical ballooning syndrome, known as Takotsubo cardiomyopathy (TCM), which is characterized by hypokinesis of the basal and middle portions of the left ventricle [2]. Although TCM may be reversible or self-limiting, it contributes significantly to morbidity and mortality after SAH, especially when it is combined with other neurogenic injuries, such as flash pulmonary edema and cardiogenic shock [3]. Furthermore, left ventricular (LV) dysfunction associated with TCM combined with cerebral vasospasm may increase the risk of delayed cerebral ischemia (DCI) [4,5], and thus it may impact clinical outcome [6]. However, the hemodynamic changes associated with post-SAH TCM are currently poorly understood, presumably because of the complicated underlying acute pathophysiological mechanisms.

Cardiovascular monitoring is essential for the diagnostic and therapeutic management of critically ill patients and for assessing LV systolic function. Echocardiography is currently the most frequently used imaging modality for bedside assessment of LVEF [7,8], but it is not ideal for real-time monitoring of systolic function because of its high intra- and interobserver variability [9]. Monitoring by transpulmonary thermodilution incorporated into the PiCCO™ monitor (PULSION Medical Systems, Munich, Germany) provides continuous cardiac output (CO) measurements derived from beat-to-beat pulse contour analysis and intermittent estimations of global end-diastolic volume (GEDV) and extravascular lung water (EVLW) [10]. Furthermore, the data can be used to calculate the cardiac function index (CFI), which is the ratio of CO to GEDV [11]. Pilot data suggest that CFI is closely related to the LV fractional area of change measured by echocardiography [12,13] and that CFI can be used to accurately assess the effects of positive inotropic therapy with dobutamine in acute circulatory failure [7]. We hypothesized that CFI could be used as a marker of LV systolic function to track the acute hemodynamic changes of post-SAH TCM.

The aims of this study were (1) to investigate the changes in cardiac function in postoperative SAH patients with TCM by transpulmonary thermodilution, and then (2) to test whether CFI and EVLW could predict outcome of TCM [14,15] in terms of DCI and modified Rankin scale (mRS).

## Material and methods

### Patients

In this retrospective analysis of a prospectively collected data set, we considered all patients enrolled in our cohort trials (Information Network Clinical Trials Registry UMIN000007509) between April 2005 and March 2013. This study was approved by the institutional review boards of the two participating stroke centers (Teine Keijinkai Hospital Institutional Review Board and Office of Research Administration at the Research Institute for Brain and Blood Vessels-Akita). According to the informed consent guidelines in Japan [16], it is unnecessary to obtain informed consent from each patient or person responsible to use secondary data from the previous database; therefore, this requirement for written informed consent was waived. Clinical, hemodynamic and radiological data of all SAH patients diagnosed with TCM and monitored with the PiCCO™ device were collected retrospectively.

### Transthoracic echocardiography

Transthoracic echocardiography was performed for all patients immediately after admission to the emergency department and was repeated simultaneously with CFI measurements using the PiCCO™ device at least more than once daily until day 14. LV ejection fraction (LVEF) was measured using the Simpson method (basically acquired using the biplane method, but a single-plane approach was used when the biplane method was technically inadequate). The echocardiographic criteria for a diagnosis of TCM included the presence of severe regional wall motion abnormalities, mostly affecting the apical and midventricular segments [4,17].

### PiCCO™-guided fluid management

Patients were managed according to previously described SAH treatment protocols (see Additional file 1) [18-21]. For hemodynamic monitoring, thermistor-tipped arterial PiCCO™ catheter (4-French, 16-cm, PV2014L16, Pulsiocath; PULSION Medical Systems) was inserted into the femoral artery and connected to the PiCCO™*plus* monitor (version 6.0; PULSION Medical Systems) for determination of CO, GEDV, CFI and EVLW in the ICU immediately after surgery. CO, GEDV and EVLW were indexed for body surface area to obtain CI, GEDI and ELWI, respectively. Full details of the PiCCO™ measurements are described elsewhere (see Additional file 1).

Hemodynamic stability was defined as CI ≥3.0 L/min/m^2^, GEDI ≥680 ml/m^2^ and ELWI ≤14 ml/kg. The upper limits chosen were the values associated with a higher risk of mortality in patients with pulmonary edema [22,23]. Hypovolemia (GEDI <680 ml/m^2^) was corrected by increasing daily intravenous fluids (or intermittent fluid infusion, as needed). Low CI associated with TCM was treated with milrinone (0.125 to 0.5 μg/kg/min, maximum) or dobutamine (3 to 9 μg/kg/min) to maintain CI ≥3.0 L/min/m^2^ [24]. Elevated ELWI (>14 ml/kg) with any signs of congestive heart failure or pulmonary edema (such as bilateral pulmonary infiltrates or cardiomegaly with a cardiothoracic ratio >50% on chest radiography) was treated with intermittent administration of furosemide (5 to 10 mg bolus) by carefully monitoring GEDI to avoid hypovolemia.

Clinical deterioration due to DCI or evidence of cerebral vasospasm on transcranial Doppler ultrasonogram (mean flow velocity in the middle cerebral artery >120 cm/s) was treated initially with volume expansion with 500 ml of 6% hydroxyethyl starch (10 ml/kg/hr, one to three times per day);if this treatment was ineffective, it was followed by hyperdynamic therapy with incremental doses of dobutamine (3 μg/kg/min, maximum of 15 μg/kg/min) or milrinone (0.125 μg/kg/min, maximum of 0.75 μg/kg/min) to raise the CI above the normal limit (>5.0 L/min/m^2^) until the resolution of symptoms [25,26].

### Measurements

The following parameters were measured at least twice daily during the study period (days 0 to 14 after the onset of SAH): CI (manufacturer’s reference range = 3 to 5 L/min/m^2^); GEDI (680 to 800 ml/m^2^); ELWI (3 to 7 ml/kg); CFI (4.5 to 6.5 min^−1^); and plasma levels of cardiac troponin I (0 to 0.5 ng/ml), adrenaline (0 to 0.1 ng/ml), noradrenaline (0.1 to 0.5 ng/ml) and brain natriuretic peptide (0 to 18.4 mg/dl). Heart rate (HR), mean arterial blood pressure (MAP), central venous pressure (CVP) and systemic vascular resistance index (SVRI) (1,700 to 2,400 dyn · s/cm^5^/m^2^) were simultaneously recorded. Measurements of transthoracic echocardiographic parameters and plasma biochemical markers were performed simultaneously with the PiCCO™ measurements at least once daily.

The primary parameter of interest was LV dysfunction (LVEF <40%, defined as mildly reduced LV systolic function where inotropic support should be considered) [14,27] between 4 and 14 days after the onset of SAH. In this study, the SAH patients were divided into two groups: LVEF <40% (TCM with LV dysfunction) and LVEF ≥40% (TCM without LV dysfunction).

DCI was defined as a new focal neurological deficit or global neurological deterioration (a decrease of two or more points on the Glasgow Coma Scale) lasting longer than 2 hours, after exclusion of intracranial hemorrhage, hydrocephalus, seizures, metabolic derangements and infection, with or without radiological signs of cerebral vasospasm [28]. In unconscious patients, DCI was diagnosed when there was a lack of neurological progress in the absence of the confounders described above or other causes of brain damage observed on imaging examinations, and if there was evidence of one of the following: cerebral vasospasm evident on transcranial Doppler ultrasound, magnetic resonance angiogram or digital subtraction angiogram; perfusion deficit on single-photon emission computed tomogram; regional cerebral hypoxia on near-infrared spectroscopy imaging scan; or cerebral infarction on an imaging study not attributable to other causes [25,29].

Functional outcome was assessed with the mRS at 3 months, divided into either poor outcome (score of 4 to 6) or favorable outcome (score of 0 to 3) [14,27]. We also recorded the following data: (1) occurrence of cardiopulmonary complications at presentation; (2) number of patients treated with hyperdynamic therapy and the clinical response (a positive response defined as at least a one-point increase in the Glasgow Coma Scale score from the worst score during the therapy, and a negative response recorded if the patient was refractory to maximal dose infusion); (3) newly diagnosed or worsening pulmonary edema after initiation of postoperative fluid therapy (day 0 to day 14), defined as ELWI >14 ml/kg based on the PiCCO™ measurements [22,23]; (4) presence of pulmonary edema after day 4 (that is, during DCI risk period); (5) daily fluid intake/output and balance; and (6) length of ICU stay.

We also considered those variables available at the time of admission: age, sex, comorbid conditions, World Federation of Neurosurgical Surgeons (WFNS) grade, modified Fisher grade and cardiopulmonary complications (for example, pulmonary edema, arrhythmia and hypotension).

### Statistical analysis

Data are expressed as the mean (standard deviation) or median (interquartile range), unless otherwise indicated. Continuous data were compared using mixed-effects analysis of variance (ANOVA; for groups and subjects over time) with the *post hoc* Bonferroni–Dunn correction, as appropriate. Univariate analyses of the relationships of categorical variables with outcomes of interest were assessed using a χ^2^ test or Fisher’s exact test. Univariate analyses of the relationships of normally distributed variables with outcomes of interest were assessed using Student’s *t*-test, and of nonnormally distributed variables were assessed using the Mann–Whitney *U* test. The variables showing significant associations with DCI and poor functional outcome on univariate analyses were entered into a multivariate logistic regression analysis. To determine the correlation between CFI and echocardiographic LVEF over time (day after SAH onset), a mixed-effects regression model was used. The ability of CFI to predict LVEF <40% was determined by receiver operating characteristic (ROC) curve analysis. The area under the curve (AUC) was calculated based on the ROC curves and expressed as a 95% confidence interval. AUC ranges from 0.5 to 1.0. An AUC closer to 1 indicates a higher predictive power. All analyses were performed using JMP Pro 11.0.0 software (SAS Institute, Cary, NC, USA). *P*-values <0.05 were considered statistically significant.

## Results

### Patient characteristics

Forty-six (8%) of the five hundred seventy-five screened patients were diagnosed with TCM. Their baseline characteristics are shown in Table 1. Patients with LV dysfunction (LVEF <40%) had a higher frequency of WFNS grade IV or V (85% versus 50%; *P* = 0.014) and a higher frequency of pulmonary edema (15% versus 50%; *P* = 0.014) at presentation than those with LVEF ≥40%. There were no significant differences between the two groups for other baseline parameters, including age, sex, modified Fisher grade based on computed tomographic scan, aneurysm location, treatment modality and hypotension.

**Table 1:** **Baseline clinical characteristics of subarachnoid hemorrhage patients with versus without left ventricular dysfunction following Takotsubo cardiomyopathy** ^**a**^

**Variable**	**LVEF ≥40% (** ***n*** **= 26)**	**LVEF <40% (** ***n*** **= 20)**	***P*** **-value**
Age, yr	67 (55 to 75)	65 (49 to 73)	0.50
Sex, females/males	18/8	14/6	0.61
WFNS grade			
I to III	13 (50%)	3 (15%)	0.014*
IV or V	13 (50%)	17 (85%)	
Modified Fisher CT grade			
2	3 (12%)	2 (10%)	0.98
3	17 (65%)	13 (65%)	
4	6 (23%)	5 (25%)	
Aneurysm location			
Anterior circulation	14 (54%)	9 (45%)	0.38
Posterior circulation	12 (46%)	11 (55%)	
Treatment			
Clipping	16 (62%)	8 (40%)	0.13
Coiling	10 (38%)	12 (60%)	
Blood biochemical marker			
Cardiac troponin T, ng/ml	0.6 (0.05 to 0.98)	1.1 (0.2 to 1.9)	0.08
Adrenaline, ng/ml	0.10 (0.03 to 0.14)	0.16 (0.09 to 0.33)	0.08
Noradrenaline, ng/ml	0.67 (0.26 to 1.15)	0.94 (0.39 to 1.52)	0.15
Brain natriuretic peptide, pg/ml	82 (32 to 130)	115 (42 to 192)	0.12
LVEF, %	44 (40 to 48)	33 (28 to 37)	0.024*
Cardiopulmonary complication			
Hypotension <90 mmHg	2 (8%)	4 (20%)	0.21
Pulmonary edema	4 (15%)	10 (50%)	0.011*

^a^CT: Computed tomography; LVEF: Left ventricular ejection fraction; WFNS: World Federation of Neurological Surgeons. Numerical variables are presented as median (interquartile range). Categorical variables are expressed as counts (percentage). Numerical variables were analyzed by Mann–Whitney *U* test or unpaired Student’s *t*-test. Categorical variables were analyzed by χ^2^ test or Fisher’s exact test. *Significant *P*-values.

### Comparison of cardiac function in patients with versus without left ventricular dysfunction

**Figure 1:**
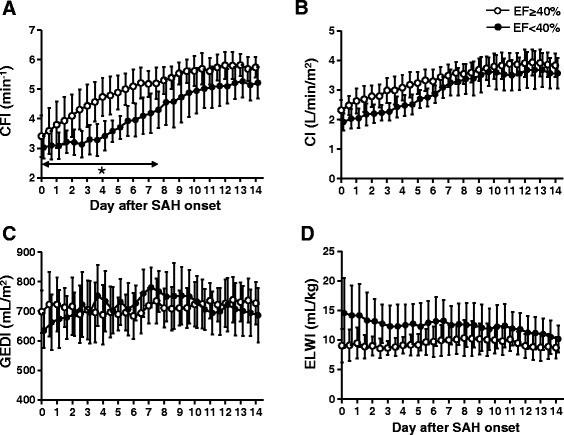
**Changes in hemodynamic parameters over 14 days in 46 subarachnoid hemorrhage patients with Takotsubo cardiomyopathy.** Cardiac function index (CFI) **(A)**, cardiac index (CI) **(B)**, global end-diastolic volume index (GEDI) **(C)** and extravascular lung water index (ELWI) **(D)** in patients with (●) or without (○) left ventricular (LV) dysfunction. Data were obtained from a total of 1,330 PiCCO™ measurements. Averaged data collected every 12 hours (twice a day) are presented. *P < 0.05 for left ventricular ejection fraction (LVEF) ≥40% versus LVEF <40% at the same time point. SAH: Subarachnoid hemorrhage.

A total of 1,330 PiCCO™ measurements were performed over a mean period of 14 ± 1 days in the 46 SAH patients with TCM. At presentation, patients in both groups (LVEF <40% and ≥40%) had low CFI (Figure 1A), mainly attributable to decreased CI (Figure 1B) induced by TCM. Low MAP (see Additional file 2) may also have been associated with the TCM-induced CI depression. The transpulmonary thermodilution-based hypovolemia (634 ± 57 ml/m^2^ versus 698 ± 70 ml/m^2^; *P* = 0.03) (Figure 1C) and pulmonary edema (14.5 ± 5.7 ml/kg versus 9.1 ± 2.7 ml/kg; *P* = 0.015) (Figure 1D) were observed only for the LVEF <40% group.

In both groups, these hemodynamic variables gradually and significantly recovered close to each normal range (multivariate ANOVA for within-group time effect; *P* < 0.0001). A statistically significant difference between the groups was detected only by CFI (multivariate ANOVA for between-groups over time effect; *P* < 0.0001). The CFI between days 0 and 7 was significantly lower in patients with LV dysfunction (LVEF <40%) than in patients with LVEF ≥40% (*P* < 0.05). No statistically significant group differences were observed for HR, MAP, CVP or SVRI throughout the study period (see Additional file 2).

### Ability of cardiac function index to detect left ventricular dysfunction

**Figure 2:**
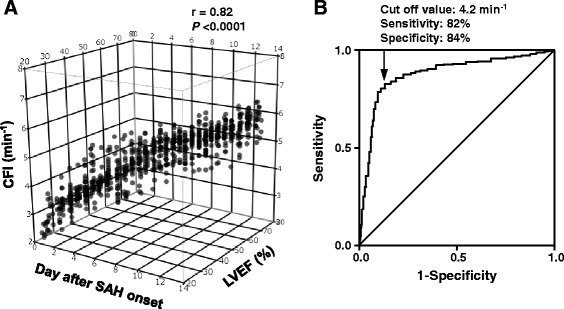
**Relationship between cardiac function index and left ventricular ejection fraction in 46 subarachnoid hemorrhage patients with Takotsubo cardiomyopathy. (A)** Three-dimensional regression data plot showing the relationship between cardiac function index (CFI) and left ventricular ejection fraction (LVEF) and time duration (days after subarachnoid hemorrhage (SAH) onset). Correlation between CFI and LVEF was analyzed by mixed-effects logistic regression (among subjects, groups, time duration). **(B)** Receiver operating characteristic curves constructed based on the sensitivity and specificity of the CFI for identifying LVEF <40%. Data were obtained from a simultaneous measurement of CFI and LVEF using the PiCCO™ device and echocardiograms, respectively (*N* = 870 data pairs).

LV dysfunction was defined by echocardiograms performed simultaneously with the PiCCO™ measurements (*N* = 870 data pairs). The transpulmonary thermodilution-derived CFI correlated significantly with echocardiographic LVEF (*r* = 0.82; *P* < 0.0001) (Figure 2A). To assess the ability of CFI to detect LVEF <40% in patients with TCM, ROC curves were generated using values averaged across all the measurements (Figure 2B). The AUC (±standard error) was 0.85 ± 0.02 (*P* < 0.001). A CFI value <4.2 min^−1^ had a sensitivity of 82% and specificity of 84% for detecting LVEF <40%.

**Figure 3:**
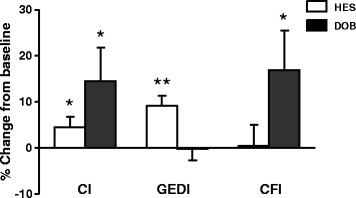
**Changes in cardiac function index during volume expansion and hyperdynamic therapy for delayed cerebral ischemia–related neurological deterioration in 25 subarachnoid hemorrhage patients with Takotsubo cardiomyopathy.** Graph shows changes in cardiac index (CI), global end-diastolic volume index (GEDI) and cardiac function index (CFI) during volume expansion with 500 ml of 6% hydroxyethyl starch (HES) (*n* = 112) or hyperdynamic therapy with dobutamine (DOB) (*n* = 55). Data were collected before and after each challenge (a 500 ml infusion of HES or an incremental infusion (3 μg/kg/min) of DOB, sampled in situations without coadministration) between days 4 and 14. *P < 0.05, **P < 0.01 compared with baseline before each challenge (% change).

We further analyzed whether CFI was useful for estimating cardiac function during hypervolemia or hyperdynamic therapy for treating DCI in 25 patients (Figure 3). CFI was not affected by mild hypervolemia (change in GEDI = 9 ± 2% from baseline; *P* < 0.01) or a slight increase in CI (change in CI = 5 ± 2% from baseline; *P* < 0.05) induced by 500 ml of 6% hydroxyethyl starch (10 ml/kg/hr). In contrast, hyperdynamic therapy with an incremental dose (3 μg/kg/min) of dobutamine (maximum dose = 9 ± 3 μg/kg/min) resulted in a change in CFI of 17 ± 9% after each dose increment (*P* < 0.05), with an overall clinical response rate of 52%.

### Impact of real-time cardiac function index values on clinical outcomes

Subgroup analysis of clinical outcomes showed that patients with LVEF <40% had a longer duration of low CFI <4.2 min^−1^ (corresponding to predicted LVEF <40%, defined by at least one CFI <4.2 min^−1^ over 24 hours) (mean difference of 4 days; *P* = 0.0001) and greater incidence of pulmonary edema after day 4 (55% versus 19%; *P* = 0.013) than patients with LVEF ≥40%, resulting in a longer ICU stay (mean difference of 2 days; *P* = 0.02) and poorer functional outcome at 3 months (mRS score of 4 to 6 = 70% versus 31%; *P* = 0.009) (Table 2). There were no significant differences between the two groups with regard to fluid or blood biochemical parameters, rate of DCI or rate of therapy-related pulmonary edema.

**Table 2:** **Variables associated with left ventricular dysfunction in subarachnoid hemorrhage patients with Takotsubo cardiomyopathy on univariate analysis** ^**a**^

**Variable**	**LVEF ≥40% (** ***n*** **= 26)**	**LVEF <40% (** ***n*** **= 20)**	***P*** **-value**
Daily fluid intake, ml	3,182 ± 564	2,976 ± 569	0.18
Fluid output, ml	2,939 ± 446	2,782 ± 541	0.21
Fluid balance, ml	282 ± 163	166 ± 108	0.09
Plasma biochemical markers (peak levels)			
Cardiac troponin T, ng/ml	1.1 (0.1 to 1.4)	2.0 (0.2 to 4.0)	0.17
Adrenaline, ng/ml	0.09 (0.03 to 0.14)	0.13 (0.04 to 0.20)	0.10
Noradrenaline, ng/ml	0.59 (0.26 to 0.76)	0.98 (0.39 to 1.66)	0.11
Brain natriuretic peptide, pg/ml	80 (21 to 132)	122 (65 to 191)	0.06
Duration of low CFI, days	2 (0 to 3)	6 (4 to 7)	0.0001*
Pulmonary edema after day 4	5 (19%)	11 (55%)	0.013*
Prevalence of DCI	5 (19%)	10 (50%)	0.029*
New or worsened pulmonary edema	2 (8%)	4 (20%)	0.22
Length of ICU stay, days	13 (10 to 14)	15 (14 to 16)	0.02*
mRS score at 3 months			
0 to 3	18 (69%)	6 (30%)	0.009*
4 to 6	8 (31%)	14 (70%)	

^a^CFI: Cardiac function index; DCI: Delayed cerebral ischemia; LVEF: Left ventricular ejection fraction. Numerical variables are presented as median (interquartile range) or mean ± standard deviation. Categorical variables are expressed as counts (percentage). Numerical variables were analyzed by Mann–Whitney *U* test or unpaired Student *t*-test. Categorical variables were analyzed by χ^2^ test or Fisher’s exact test. Pulmonary edema was defined as ELWI >14 ml/kg based on the PiCCO™ measurements. Duration of low CFI was defined by at least one CFI <4.2 min^−1^ (corresponding to predicted LVEF <40%) over 24 hours. *Significant *P*-values.

Multivariate logistic regression analysis including significant factors associated with DCI (WFNS grades IV and V, duration of low CFI and coexisting pulmonary edema) and functional outcomes (WFNS grades IV and V, prevalence of DCI, duration of low CFI, coexisting pulmonary edema and length of ICU stay) on univariate analysis (see Additional files 3 and 4) showed that duration of low CFI (<4.2 min^−1^) was independently associated with DCI (odds ratio (OR) = 2.14, 95% confidence interval = 1.33 to 2.84; *P* = 0.004) and poor 3-month functional outcome on mRS score of 4 to 6 (OR = 1.87, 95% confidence interval = 1.06 to 3.29; *P* = 0.02) (Table 3). Coexisting pulmonary edema (ELWI >14 ml/kg) also increased the risk of poor functional outcome at 3 months (OR = 2.10, 95% confidence interval = 1.11 to 3.97; *P* = 0.04).

**Table 3:** **Variables associated with delayed cerebral ischemia and functional outcome in 46 subarachnoid hemorrhage patients with Takotsubo cardiomyopathy on multivariate analysis** ^**a**^

**Covariates**	**Odds ratio (95% confidence interval)**	***P*** **-value**
DCI		
WFNS grade IV or V	1.64 (0.07 – 5.87)	0.70
Duration of low CFI, days	2.14 (1.33 – 2.84)	0.004*
Coexist pulmonary edema (ELWI >14 ml/kg) after day 4	1.66 (0.32 – 2.21)	0.36
Poor functional outcome at 3-month follow-up		
WFNS grade IV or V	1.17 (0.46 – 3.76)	0.20
DCI	1.68 (0.07 – 7.29)	0.39
Duration of low CFI, days	1.87 (1.06 – 3.29)	0.02*
Pulmonary edema after day 4	2.10 (1.11 – 3.97)	0.04*
Length of ICU stay, days	1.93 (0.54 – 1.41)	0.59

^a^CFI: Cardiac function index; DCI: Delayed cerebral ischemia; ELWI: Extravascular lung water index; WFNS: World Federation of Neurological Surgeons. Poor functional outcome was defined as modified Rankin Scale scores of 4 to 6. Pulmonary edema was defined as ELWI >14 ml/kg based on the PiCCO™ measurements. Duration of low CFI was defined by at least one CFI <4.2 min^−1^ (corresponding to predicted LVEF <40%) over 24 hours. *Significant *P*-values.

## Discussion

Our present detailed analysis of hemodynamic parameters monitored using an advanced transpulmonary thermodilution technique with the PiCCO™ system provides new evidence regarding cardiac function and volumetric changes in patients with SAH complicated by TCM. This study shows that CFI measured by transpulmonary thermodilution provides a reliable real-time estimation of LV systolic function in patients with TCM. CFI in our patients behaved as an index of LV systolic function [7,13] in that (1) it had significant correlation with the echocardiographic LVEF, which is considered the gold standard for estimating LV systolic function, and reliably tracked treatment-induced changes in LVEF; and (2) it did not change with fluid loading, but increased after administration of an inotropic agent for hyperdynamic therapy. Furthermore, CFI was sensitive for detecting LVEF <40%, which is the point at which inotropic support should be considered [14,27].

Several comorbid risk factors predictive of SAH-induced TCM and neurogenic pulmonary edema may operate via increased catecholamines [30,31]. Unfortunately, differences in the levels of circulating catecholamines or other biochemical and/or hemodynamic markers related to stress and fluid regulation did not have a major impact on outcomes of patients with TCM with or without LV dysfunction. The results of a recent multicenter cohort study suggest that low CI, decreased GEDI or increased systemic vascular resistance (that is, afterload mismatch) at an early stage may be associated with DCI in patients with poor functional outcomes [32], but the prognostic factors in patients with TCM are still unclear. Our results suggest that TCM-related low CFI (<4.2 min^−1^) lasting 4 days or more should alert the physician that LV contraction is probably impaired and that there is an increased risk of DCI and poor functional outcome. Persistent LV dysfunction has also been reported to be associated with pulmonary edema [33], indicating that early assessment of ELWI and other clinical and radiological data is useful for early detection of pulmonary edema and the need for detailed examination (such as differentiating between hydrostatic and permeability edema to evaluate acute lung injury and/or acute respiratory distress syndrome) [23,34,35].

Our data demonstrate that duration of LV dysfunction (CFI <4.2 min^−1^ to predict LVEF <40% with sensitivity of 82% and specificity of 84%) and concurrent pulmonary edema during DCI risk period (days 4 to 14 after SAH) are associated with prognosis in patients with post-SAH TCM. Transient LV dysfunction is a well-recognized complication of SAH and is often referred to as neurogenic stunned myocardium [33,36]. The results of a recent analysis of pooled data suggest that TCM and neurogenic stunned myocardium, regardless of whether it has regional wall motion abnormalities and elevation of cardiac enzyme levels, are likely to have common underlying mechanisms and pathological processes inducing cardiomyopathy [33]. On the other hand, a clinical entity with milder forms of TCM that do not progress to LV dysfunction [36] and less prevalence of pulmonary edema in TCM without LV dysfunction [15,33] has also been described [36]. These data all highlight the importance of real-time CFI and ELWI monitoring with transpulmonary thermodilution, particularly in postoperative SAH patients with TCM, at least during the DCI risk period.

Although we selected postoperative patients with post-SAH TCM from our prospective cohort of consecutive SAH patients, this study is limited by its relatively small sample size and retrospective nature. The study is also limited by selection bias because it included only patients who survived long enough to undergo aneurysm coiling or clipping. The prevalence of TCM in our patients was comparable with previously reported data (4% to 15%) [4,15,37]. This study has the advantage of including the largest reported number of patients with post-SAH TCM who were successfully monitored for cardiac performance and volume status for approximately 14 days. The findings of this two-center study should be validated by larger cohort trials. As use of catecholamines for the treatment of DCI in patients with TCM is still controversial [5,38,39], future studies should also be focused on establishing optimal methods of fluid management in these patients.

## Conclusions

The results of this study suggest that prolonged cardiac dysfunction and concurrent pulmonary edema contribute to poor functional outcome in SAH patients with TCM. Serial measurements of transpulmonary thermodilution-derived CFI and ELWI may provide an easy bedside method of estimating changes in LV systolic function and EVLW and predicting clinical outcome.

## Key messages

- The incidence of TCM in treatable SAH patients was 8%, and half of them had concurrent pulmonary edema.
- Prolonged cardiac dysfunction and pulmonary edema associated with TCM increased the risk of DCI, contributing to poor functional outcome in SAH patients.
- Serial measurement of CFI and ELWI by transpulmonary thermodilution may provide an easy bedside method of detecting early changes in TCM-induced cardiopulmonary function to direct proper post-SAH treatment.

## Additional files

Additional file 1:
**Detailed description of general managements and PiCCO™ measurements.**
Additional file 2:
**Changes in hemodynamic parameters over 14 days in 46 SAH patients with TCM.** **(A)** Heart rate (HR), **(B)** Mean arterial blood pressure (MAP), **(C)** Central venous pressure (CVP) and **(D)** Systemic vascular resistance index (SVRI), each in patients with (●) versus without (○) left ventricular dysfunction. Data were obtained from a total of 1,330 PiCCO™ measurements. Averaged data collected every 12 hours (twice a day) have been presented. **P* < 0.05 for LVEF ≥40% versus LVEF <40% at the same time point. Additional file 3:
**Univariate analysis of DCI in 46 SAH patients with TCM.**
Additional file 4:
**Univariate analysis of functional outcome at 3-month follow-up in 46 SAH patients with TCM.**

## Abbreviations

AUC: Area under the curve
CFI: Cardiac function index
CI: Cardiac index
CO: Cardiac output
CVP: Central venous pressure
DCI: Delayed cerebral ischemia
ELWI: Extravascular lung water index
EVLW: Extravascular lung water
GEDI: Global end-diastolic volume index
GEDV: Global end-diastolic volume
HR: Heart rate
LV: Left ventricular
LVEF: Left ventricular ejection fraction
MAP: Mean arterial blood pressure
mRS: Modified Rankin Scale
OR: Odds ratio
ROC: Receiver operating characteristic
SAH: Subarachnoid hemorrhage
SVRI: Systemic vascular resistance index
TCM: Takotsubo cardiomyopathy
WFNS: World Federation of Neurosurgical Surgeons
