# Supplementary material for: Impact of transpulmonary thermodilution-based cardiac contractility and extravascular lung water measurements on clinical outcome of patients with Takotsubo cardiomyopathy after subarachnoid hemorrhage: a retrospective observational study
Source: Crit Care. 2014 Aug 12;18(4):482. doi: 10.1186/s13054-014-0482-4 (PMC4243958; doi:10.1186/s13054-014-0482-4)
Supplement: Additional file 1: — Detailed description of general managements and PiCCO™ measurements. [file 13054_2014_482_MOESM1_ESM.pdf]

Additional file 1

Methods

General management

All aneurysms were treated under general anesthesia [1]. Induction of anesthesia was performed with propofol by setting the target concentration to 3 μg/mL using a target-controlled infusion system, followed by remifentanil. Tracheal intubation was facilitated by vecuronium and immediately after the induction of anesthesia, a 7 Fr. central venous catheter was inserted into the femoral vein for fluid and drug administration. Anesthesia was maintained with continuous infusion of remifentanil and propofol. During this period, patients were maintained with an intravenous crystalloid infusion of 1–2 mL/kg/h to maintain euvolemia based on the fluid balance, calculated from the difference between total water intake and loss at least every 30-min by an anesthesiologist. Postoperatively, all patients were transferred to the intensive care unit after confirmation of spontaneous ventilation. Tracheal extubation was performed when body temperature was > 36ºC, breathing was spontaneous with adequate blood gas variables, and hemodynamics were stable.

For postoperative fluid management, the patients received a baseline crystalloid infusion of 1,500–3,000 mL/d and supplemental fluid/drug administration up to day 14, in accordance with PiCCO™-guided fluid therapy [2-6]. They were rested in bed without sedation and with intravenous fluids and oral food intake if possible. Intracranial hypertension was treated with cerebrospinal drainage. Hyponatremia (defined as a serum sodium level of < 135 mEq/L for at least 2 consecutive days) was corrected by adding an ampule(s) of 10% NaCl (20 mL) to the main fluid bag. If hyponatremia persisted, fludrocortisone (0.3 mg/d) was given as necessary [7]. Blood transfusion was performed only when the hematocrit level was < 30%. Patients received a bolus injection of nicardipine if the systolic blood pressure increased to > 180 mmHg. Nimodipine was not used as this drug is unavailable in Japan. Fasudil hydrochloride was administered three times a day as an intravenous dose of 30 mg at each administration [8].

Single-indicator transpulmonary thermodilution technique using PiCCO^{TM} system

The single-indicator transpulmonary thermodilution system incorporated into the PiCCO^{TM}*plus* monitor (version 6.0; Pulsion Medical Systems, Munich, Germany) measures the change in temperature over time induced by a bolus injection of cold saline.[9] A thermistor-tipped arterial PiCCO^{TM} catheter (Pulsiocath: 4 Fr, 16 cm, PV2014L16; Pulsion Medical Systems) was inserted into the brachial artery and connected to the PiCCO^{TM} monitor in the intensive care unit ICU immediately after surgery. PiCCO^{TM} measurements were obtained by triplicate injections of 15-mL boluses of ice-cold saline via the central venous line. Based on the thermodilution curve, the PiCCO^{TM} system calculated the cardiac output (CO) using the modified Stewart–Hamilton equation, the mean transit time, and the exponential downslope time of the curve. Intrathoracic thermal volume was calculated as the product of CO and mean transit time, pulmonary thermal volume was calculated as the product of CO and exponential downslope time, and stroke volume was calculated as CO divided by heart rate. Global end-diastolic volume (GEDV) was calculated as the difference between intrathoracic thermal volume and pulmonary thermal volume, representing the volume of blood in the four heart chambers. Cardiac function index (CFI) was defined as the ratio of CO to GEDV. Extravascular lung water (EVLW) was calculated from GEDV using an algorithm established from data obtained from earlier double-indicator transpulmonary thermodilution using indocyanine green [9].

The PiCCO^{TM} system operates in such a way that every time a thermodilution injection is performed, the pulse contour analysis is automatically and immediately self-calibrating from the shape of the arterial pressure wave with the new value of transpulmonary thermodilution to compute each single stroke volume (SV) [10]. As pulse contour analysis continuously measures SV and arterial pressure, cardiac output (CO = SV × heart rate) and systemic vascular resistance (SVR = mean arterial pressure − central venous pressure × 79.9/CO) are computed simultaneously and displayed for continuous monitoring. The CO, GEDV, EVLW, SV, and SVR

were indexed to body surface area by means of the DuBois formula (body weight [kg] × body length [cm]$^{0.725}$ × 71.84), yielding the cardiac index (CI, manufacture's normal range: 3.0–5.0 L/min/m^2), GEDV index (GEDI, 680–800 mL/m^2), EVLW index (ELWI, 3–7 mL/kg), CFI (4.5–6.5 min^{-1}), and SVR index (SVRI, 1700–2400 dyn·s/cm^5/m^2).

References

1. Mutoh T, Ishikawa T, Nishino K, Yasui N: **Evaluation of the FloTrac™ uncalibrated continuous cardiac output system for perioperative hemodynamic monitoring after subarachnoid hemorrhage.** *J Neurosurg Anesthesiol* 2009, **21:**218-225.
2. Mutoh T, Kazumata K, Terasaka S, Taki Y, Suzuki A, Ishikawa T: **Early intensive versus minimally invasive approach to postoperative hemodynamic management after subarachnoid hemorrhage.** *Stroke* 2014, **45:**1280-1284.
3. Mutoh T, Kazumata K, Yokoyama Y, Ishikawa T, Taki Y, Terasaka S, Houkin K: **Comparison of postoperative volume status and hemodynamics between surgical clipping and endovascular coiling in patients after subarachnoid hemorrhage.** *J Neurosurg Anesthesiol* 2014, in press.
4. Mutoh T, Kazumata K, Ajiki M, Ushikoshi S, Terasaka S: **Goal-directed fluid management by bedside transpulmonary hemodynamic monitoring after subarachnoid hemorrhage.** *Stroke* 2007, **38:**3218-3224.
5. Mutoh T, Kazumata K, Kobayashi S, Terasaka S, Ishikawa T: **Serial measurement of extravascular lung water and blood volume during the course of neurogenic pulmonary edema after subarachnoid hemorrhage: initial experience with 3 cases.** *J Neurosurg Anesthesiol* 2012, **24:**203-208.
6. Mutoh T, Kazumata K, Ishikawa T, Terasaka S: **Performance of bedside transpulmonary thermodilution monitoring for goal-directed hemodynamic management after subarachnoid hemorrhage.** *Stroke* 2009, **40:**2368-2374.

7. Moro N, Katayama Y, Kojima J, Mori T, Kawamata T: **Prophylactic management of excessive natriuresis with hydrocortisone for efficient hypervolemic therapy after subarachnoid hemorrhage.** *Stroke* 2003, **34:**2807-2811.

8. Shibuya M, Suzuki Y, Sugita K, Saito I, Sasaki T, Takakura K, Nagata I, Kikuchi H, Takemae T, Hidaka H, et al.: **Effect of AT877 on cerebral vasospasm after aneurysmal subarachnoid hemorrhage. Results of a prospective placebo-controlled double-blind trial.** *J Neurosurg* 1992, **76:**571-577.

9. Sakka SG, Reuter DA, Perel A: **The transpulmonary thermodilution technique.** *J Clin Monit Comput* 2012, **26:**347-353.

10. Gödje O, Höke K, Goetz AE, Felbinger TW, Reuter DA, Reichart B, Friedl R, Hannekum A, Pfeiffer UJ: **Reliability of a new algorithm for continuous cardiac output determination by pulse-contour analysis during hemodynamic instability.** *Crit Care Med* 2002, **30:**52-58.
