# Supplementary figures and images for: Impact of transpulmonary thermodilution-based cardiac contractility and extravascular lung water measurements on clinical outcome of patients with Takotsubo cardiomyopathy after subarachnoid hemorrhage: a retrospective observational study
Source: Crit Care. 2014 Aug 12;18(4):482. doi: 10.1186/s13054-014-0482-4 (PMC4243958; doi:10.1186/s13054-014-0482-4)

**A**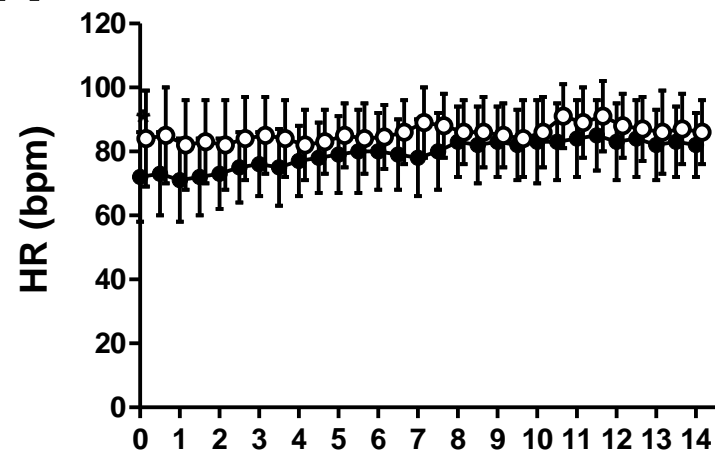**B**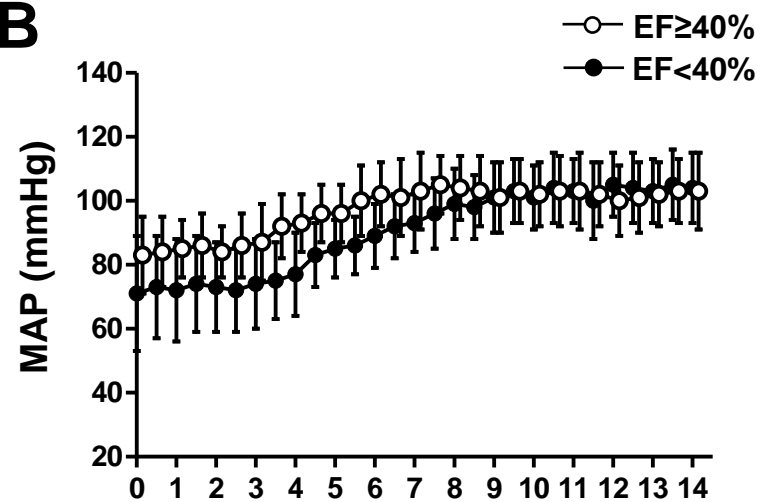**C**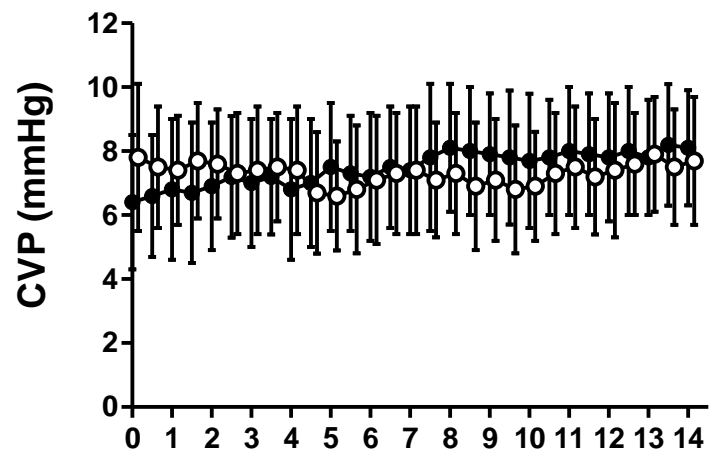**D**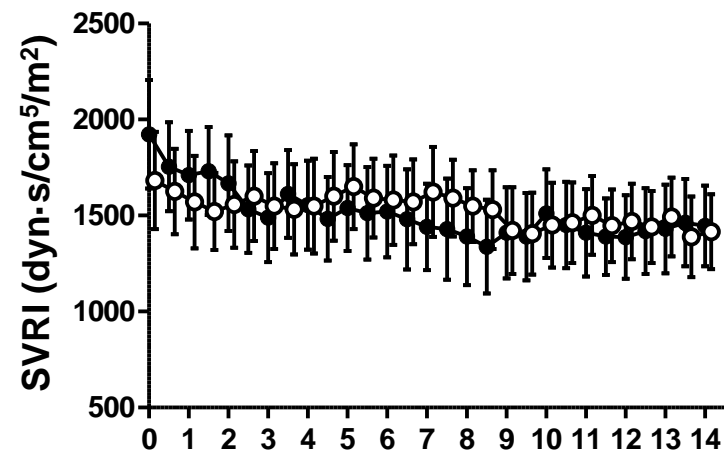

Supplement: Additional file 2: — Changes in hemodynamic parameters over 14 days in 46 SAH patients with TCM. (A) Heart rate (HR), (B) Mean arterial blood pressure (MAP), (C) Central venous pressure (CVP) and (D) Systemic vascular resistance index (SVRI), each in patients with (●) versus without (○) left ventricular dysfunction. Data were obtained from a total of 1,330 PiCCO™ measurements. Averaged data collected every 12 hours (twice a day) have been presented. *P < 0.05 for LVEF ≥40% versus LVEF <40% at the same time point. [file 13054_2014_482_MOESM2_ESM.pdf]
