# Supplementary material for: Impact of transpulmonary thermodilution-based cardiac contractility and extravascular lung water measurements on clinical outcome of patients with Takotsubo cardiomyopathy after subarachnoid hemorrhage: a retrospective observational study
Source: Crit Care. 2014 Aug 12;18(4):482. doi: 10.1186/s13054-014-0482-4 (PMC4243958; doi:10.1186/s13054-014-0482-4)
Supplement: Additional file 3: — Univariate analysis of DCI in 46 SAH patients with TCM. [file 13054_2014_482_MOESM3_ESM.pdf]

Additional file3

Variables associated with DCI in SAH patients with TCM on univariate analysis

**Covariates**	**DCI negative (*n* = 31)**	**DCI positive (*n*=15)**	**Odds Ratio (95% CI)**	***P***
Age (years)	68 (55-74)	64 (49-73)	N/A	0.08
Sex (female)	21 (68%)	11 (73%)	1.3 (0.7–2.3)	0.54
Modified Fisher grade 3-4	27 (87%)	14 (93%)	2.0 (1.1–3.9)	0.24
WFNS grade IV-V	17 (55%)	13 (87%)	5.4 (1.0–27.8)	0.049*
Daily CFI (min^{-1}) day 0 to day 3	3.5 (3.2-3.7)	3.2 (2.9-3.5)	N/A	0.07
Daily CFI (min^{-1}) day 4 to day 14	4.4 (3.9-4.8)	4.0 (3.6-4.5)	N/A	0.064
Duration of low CFI (day)	2 (0-3)	5 (4-7)	N/A	<0.0001*
Coexist pulmonary edema after day 4	6 (19%)	10 (67%)	8.3 (2.1 – 33.6)	0.003*
Length of ICU stay (day)	13 (10-14)	14 (12-16)	N/A	0.07

Numerical variables were presented as median (interquartile range). Categorical variables were expressed as counts (percentage). Numerical variables were analyzed by Mann-Whitney *U* test or unpaired Student *t* test. Categorical variables were analyzed by chi-square test or Fisher exact test. Daily CFI, minimum daily values were collected during early phase (day 0 to day 3) or during DCI risk period (day 4 to day 14), and compared the values between the groups. Duration of low CFI was defined by at least one CFI <4.2 min^{-1} (corresponding to *predicted* LVEF <40%) over 24 hours. N/A, not available. *Significant *P* values.
