# Supplementary material for: Impact of transpulmonary thermodilution-based cardiac contractility and extravascular lung water measurements on clinical outcome of patients with Takotsubo cardiomyopathy after subarachnoid hemorrhage: a retrospective observational study
Source: Crit Care. 2014 Aug 12;18(4):482. doi: 10.1186/s13054-014-0482-4 (PMC4243958; doi:10.1186/s13054-014-0482-4)
Supplement: Additional file 4: — Univariate analysis of functional outcome at 3-month follow-up in 46 SAH patients with TCM. [file 13054_2014_482_MOESM4_ESM.pdf]

Additional file4

Variables associated with functional outcome at 3-month follow-up in SAH patients with TCM on univariate analysis

**Covariates**	**Good mRS (*n*=24)**	**Poor mRS (*n*=22)**	**Odds Ratio (95% CI)**	***P***
Age	67 (55-75)	65 (49-72)	N/A	0.17
Female	17 (71%)	15 (68%)	1.1 (0.6–2.0)	0.88
Modified Fisher grade 3-4	21 (88%)	20 (91%)	1.4 (0.6–3.4)	0.65
WFNS grade IV-V	12 (50%)	18 (82%)	4.5 (1.2–17.3)	0.03*
Prevalence of DCI	4 (17%)	11 (50%)	5.0 (1.3–19.5)	0.027*
Minimum CFI (min^{-1}) day 0 to day 3	3.5 (3.1-3.8)	3.2 (2.9-3.5)	N/A	0.07
Minimum CFI (min^{-1}) day 4 to day 14	4.5 (4.2-4.8)	4.1 (3.6-4.6)	N/A	0.10
Duration (day) of low CFI (<4.2 min^{-1})	2 (0-3)	4 (3-6)	N/A	<0.0001*
Coexist pulmonary edema after day 4	4 (18%)	12 (55%)	5.4 (1.4 – 21.3)	0.03*
Length of ICU stay (day)	13 (10-15)	15 (14-16)	N/A	0.019*

Numerical variables were presented as median (interquartile range). Categorical variables were expressed as counts (percentage). Numerical variables were analyzed by Mann-Whitney *U* test or unpaired Student *t* test. Categorical variables were analyzed by chi-square test or Fisher exact test. Functional outcome was assessed by modified Rankin scale (good, mRS score of 0-3; poor, mRS score of 4-6). Daily CFI, minimum daily values were collected during early phase (day 0 to day 3) or during DCI risk period (day 4 to day 14), and compared the values between the groups. Duration of low CFI was defined by at least one CFI <4.2 min^{-1} (corresponding to *predicted* LVEF <40%) over 24 hours. N/A, not available. *Significant *P* values.
